# A radiomics nomogram based on MSCT and clinical factors can stratify fibrosis in inflammatory bowel disease

**DOI:** 10.1038/s41598-023-51036-w

**Published:** 2024-01-12

**Authors:** Xu Zeng, Huijie Jiang, Yanmei Dai, Jin Zhang, Sheng Zhao, Qiong Wu

**Affiliations:** Department of Radiology, The Second Affiliated Hospital of Harbin Medical University, No.246 Xuefu Road, Nangang District, Harbin, 150081 Helongjiang Province People’s Republic of China

**Keywords:** Inflammatory bowel disease, Crohn's disease, Ulcerative colitis

## Abstract

Intestinal fibrosis is one of the major complications of inflammatory bowel disease (IBD) and a pathological process that significantly impacts patient prognosis and treatment selection. Although current imaging assessment and clinical markers are widely used for the diagnosis and stratification of fibrosis, these methods suffer from subjectivity and limitations. In this study, we aim to develop a radiomics diagnostic model based on multi-slice computed tomography (MSCT) and clinical factors. MSCT images and relevant clinical data were collected from 218 IBD patients, and a large number of quantitative image features were extracted. Using these features, we constructed a radiomics model and transformed it into a user-friendly diagnostic nomogram. A nomogram was developed to predict fibrosis in IBD by integrating multiple factors. The nomogram exhibited favorable discriminative ability, with an AUC of 0.865 in the validation sets, surpassing both the logistic regression (LR) model (AUC = 0.821) and the clinical model (AUC = 0.602) in the test set. In the train set, the LR model achieved an AUC of 0.975, while the clinical model had an AUC of 0.735. The nomogram demonstrated superior performance with an AUC of 0.971, suggesting its potential as a valuable tool for predicting fibrosis in IBD and improving clinical decision-making. The radiomics nomogram, incorporating MSCT and clinical factors, demonstrates promise in stratifying fibrosis in IBD. The nomogram outperforms traditional clinical models and offers personalized risk assessment. However, further validation and addressing identified limitations are necessary to enhance its applicability.

## Introduction

Inflammatory bowel disease (IBD), which includes Crohn's disease (CD) and ulcerative colitis (UC), is a chronic and relapsing inflammatory disorder of the gastrointestinal tract^1,2^. The global incidence of inflammatory bowel disease (IBD) increased by 47.45%, from an estimated 3.32 million cases to 4.90 million cases between 1990 and 2019, which poses significant impacts in patients' quality of life and challenges in terms of diagnosis, treatment, and management^3–7^. One of the key complications of IBD is the development of fibrosis, a pathological process characterized by excessive accumulation of extracellular matrix components in the affected intestinal wall^8^. Fibrosis can lead to structural alterations, strictures, and functional impairments, ultimately contributing to disease progression and complications^9^.

Accurate assessment and stratification of fibrosis in IBD patients are crucial for determining appropriate treatment strategies and optimizing patient outcomes. While conventional imaging modalities, such as magnetic resonance imaging (MRI) and computed tomography (CT), have been used to evaluate fibrosis in IBD, their limitations in providing quantitative and objective measurements have prompted the exploration of alternative approaches^10^. Radiomics, a rapidly evolving field in medical imaging, has gained significant attention in various clinical domains, where it has shown great potential for predicting treatment response, prognosis, and even guiding personalized therapies^11^. Radiomics refers to the extraction of a large number of quantitative imaging features from medical images, followed by the application of advanced data analysis techniques^12,13^. These features capture the heterogeneity and spatial patterns of tissues, enabling a more comprehensive and objective characterization of disease processes^14^. It has been used in the identification of pre-therapeutic predictive markers for response and prognosis in individualized patient treatment for gastric cancer^15^, colorectal cancer^16^, liver cancer^17^, and other digestive disorders^18^. In the context of IBD, radiomics-based approaches have emerged as a valuable tool for assessing disease activity^19^, distinguishing between active inflammation and fibrosis^20^, and predicting treatment response^21^.

Especially, a study demonstrated that semi-automated measurements of structural bowel damage, including bowel wall thickness, dilation, and lumen diameter, are highly comparable to those taken by experienced radiologists, with similar accuracy in detecting intestinal strictures^22^. Another multicenter, retrospective study used a machine learning-based radiomic model and demonstrated superior performance to radiologists in accurately predicting intestinal fibrosis^18^. Moreover, research also revealed that apparent diffusional kurtosis could effectively differentiate between no or mild fibrosis and moderate to severe fibrosis in CD patients, achieving a high sensitivity of 95.9% and a specificity of 78.1%. This indicates the potential of apparent diffusional kurtosis as a valuable MRI imaging tool for evaluating bowel fibrosis. However, most of these current studies employing radiomics have been limited to traditional CT and MRI scans, with patient cohorts typically smaller than 200. Considering the superior advantages of Multi-Slice Computed Tomography (MSCT) over traditional CT, such as faster imaging of larger body areas and higher spatial resolution for detecting fine details, MSCT could potentially reveal more intricate imaging features in IBD fibrosis. Therefore, it's essential to establish a comprehensive and reliable radiomics model based on MSCT, specifically designed for stratifying fibrosis in IBD patients, in a larger population. This remains an active area of research, with MSCT's advanced capabilities offering promising avenues for improved diagnostic accuracy.

This study aims to address this research gap by proposing a radiomics nomogram based on MSCT and clinical factors for 218 IBD patients. By combining quantitative imaging features extracted from MSCT scans with relevant clinical parameters, we aim to develop a robust and user-friendly tool that can accurately stratify fibrosis in IBD patients. The nomogram will enable clinicians to make informed decisions regarding treatment selection, surgical planning, and disease monitoring, ultimately leading to enhance precision and effectiveness of fibrosis assessment in IBD patients.

## Methods

### Participants

**Figure 1:**
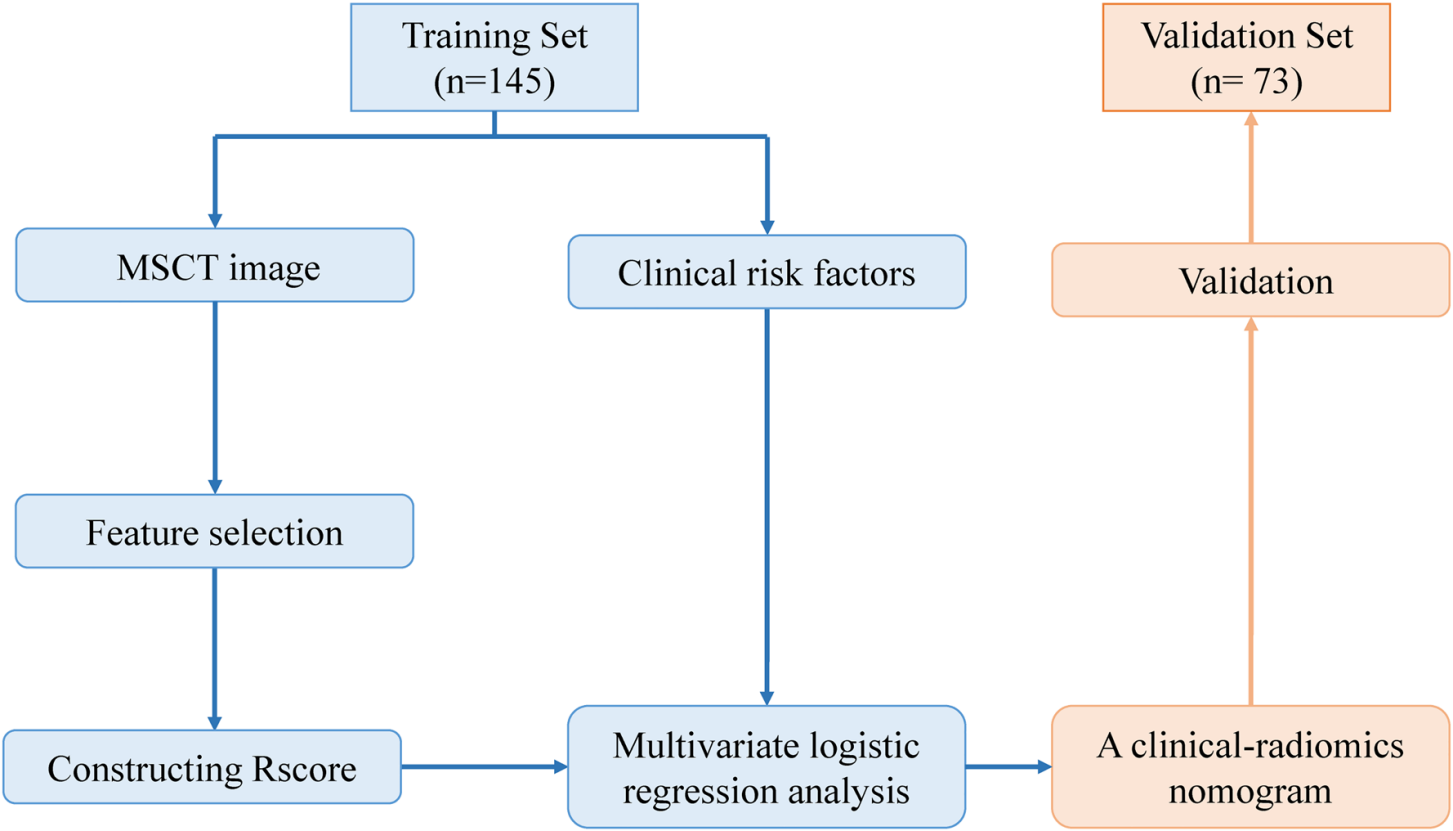
The process in the development of the clinical-radiomics nomogram for predicting the risk of fibrosis in inflammatory bowel disease.

This study was conducted in accordance with the Declaration of Helsinki guidelines and approved by The Second Affiliated Hospital of Harbin Medical University. Informed consent was obtained from all participants in this study. Clinical data from IBD patients treated at The Second Affiliated Hospital of Harbin Medical University, between June 2015 and October 2022 were collected. In terms of original images, the quality control was conducted following the guidelines for imaging examination and reporting of IBD in China. Inclusion criteria consisted of patients who have been diagnosed as CD or UC^23^, and have the qualification of MSCT images. Exclusion criteria included pregnant or nursing women, hyperthyroidism or iodine allergy, severe diseases affecting vital signs, mental illness or low cognitive ability, liver diseases, kidney diseases, digestive tract cancer, unqualified images, and images outside the colon and rectum. A total of 218 IBD patients (113 CD, 105 UC) who underwent both MSCT enhancement scans and endoscopy were included in the study. The dataset was divided into training data (n = 145) and test data (n = 73) using a random split method (2:1 ratio) (Fig. 1).

### Image data

Prior to the CT examination, patients followed a diet without solid food. They abstained from eating for 8 h prior to the examinations and the laxatives were taken beforehand. Additionally, they consumed 2000–3000 mL of 2.5% isotonic mannitol in intervals of 400–500 mL every 15 min.

For the enhanced scanning procedure, 256-detector rows CT machine was used. From the diaphragmatic crest to the pubic symphysis, a 5 mm thick scan was performed along with a 0.75 mm thick thin layer reconstruction. A contrast medium was administered through a high-pressure syringe inserted into the anterior cubital vein. A dosage of 60–80 mL of the contrast medium containing 300 mg/mL of iodine was injected at a rate of 3.0–3.5 ml/s. After contrast injection, an arterial phase scan (25–35 s after) and an intravenous phase scan (65–90 s after) were performed.

## Reference standard for intestinal fibrosis

We obtained histological tissue samples through endoscopic biopsies or surgical resections performed at our hospitals. Histological sections obtained from our hospitals were subjected to evaluation by a skilled pathologist who specialized in bowel pathology for 15 years. The pathologist, blinded to the clinical and radiological data, applied consistent criteria to assess the degree of bowel fibrosis using Masson's trichrome staining and bowel inflammation using H&E staining. A semi-quantitative scoring system was employed to assign scores ranging from 0 to 4, representing the severity of fibrosis and inflammation, with 0 indicating no fibrosis/inflammation and 4 indicating severe fibrosis/inflammation^18^. These scores facilitated the categorization of fibrosis and inflammation into two groups: none-mild (scores 0–2) and moderate-severe (scores 3–4).

### Image segmentation and feature extraction

An original DICOM image was analyzed for radiomics features based on a two-dimensional region of interest (ROI), which was meticulously delineated by an experienced abdominal radiologist using manual segmentation on the axial slices of the MSCT images. The radiologist conducted this process while blinded to the clinical and pathological data. Care was taken to include the entire thickness of the bowel wall in the ROI, while excluding adjacent fat and vascular structures. This delineation was performed using ITK-SNAP software (www.itksnap.org), ensuring precision and consistency across all cases.

For the image resampling process, we employed the B-spline interpolation method, setting the target resolution to 1mm x 1mm x 1mm. The order of interpolation was set at 3, providing a balance between smoothness and accuracy in the resampling results. Various image preprocessing methods were applied, such as gray level co-occurrence matrix (GLCM), gray level run length matrix (GLRLM), gray level size zone matrix (GLSZM), and gray level dependence matrix (GLDM). To be specific, we quantized the image grayscale values into 64 discrete levels. For the application of various image preprocessing methods, a window size of 5mm x 5mm was utilized. Specifically, for the extraction of Gray Level Co-occurrence Matrix (GLCM) features, a pixel pair distance of 1 was set, and features were calculated in all four principal directions (0°, 45°, 90°, and 135°). At last, a total of 1,450 standardized radiomic features were extracted.

The features were named by the categorization structured in three distinct levels: the initial level detailed the image preprocessing method and associated parameters, for instance, log-sigma-1-0-mm. Subsequently, the second level denoted the type of feature, encompassing options such as first-order, sphericity, and GLDM. Finally, the third level pinpointed the precise feature extraction method, like run length non-uniformity, ensuring a comprehensive and systematic representation of the process.

The extraction process utilized PyRadiomics, an open-source platform implemented in Python for processing and extracting radiomic features from medical images^24–26^.

### Feature selection and radiomics score construction

To ensure proper model evaluation, the dataset was divided into training (n = 145) and testing (n = 73) sets. Feature selection was performed using various machine learning algorithms, including Logistic Regression (LR), Support Vector Machine (SVM), Random Forest (RF), Stochastic Gradient Descent (SGD), and Linear Discriminative Analysis (LDA). Specifically, we employed LR with an L1 regularization parameter finely set at 0.01. Simultaneously, a SVM with a linear kernel was utilized, and its regularization parameter (C) was calibrated at 1. The RF algorithm played a crucial role as well, configured with 100 trees and a maximum depth of 10. Additionally, SGD was applied, operating under a logistic loss function and a learning rate set at 0.01. Lastly, LDA was implemented, primarily focusing on finding the most discriminating linear combination of features, and operated with its default parameter settings.

To identify the most relevant and informative features from these algorithms for constructing the radiomics score (Rscore), we first gathered all the features selected by different machine learning algorithms into one pool. We then assigned weights to these features based on how often they were selected across the algorithms, giving more importance to those chosen frequently. After normalizing the weights to ensure they added up to one, we calculated the radiomics score for each data instance by multiplying each feature’s value by its weight and summing these products. Finally, we checked the accuracy of these scores using our testing dataset, making adjustments to the weights as needed to optimize performance.

### Development of the clinical-radiomics nomogram

To incorporate relevant clinical information, the clinical factors were selected based on their relevance and significance to the outcome of interest, as established through a comprehensive review of existing literature^27–29^. We identified eight clinical factors that were potentially associated with the outcome of interest.

To determine their significance, single-factor analysis was performed, comparing the fibrosis or non-fibrosis in IBD. Factors showing significant differences were considered for further analysis and inclusion in the nomogram. The clinical and radiomic features were combined in a multivariate LR analysis to construct the nomogram.

The regression coefficients derived from the analysis were used to assign weights to each feature in the nomogram. Features with higher absolute values of coefficients were assigned higher weights, reflecting their stronger influence on the outcome. By summing the weighted scores of the selected features, the nomogram provided a personalized risk estimation for each patient. In detail, each patient’s values for the selected predictors were input into the nomogram, and the corresponding weighted scores were summed up to obtain a total score. This total score was then translated into a probability of the outcome, using the logistic function. This process allows for individualized risk estimation, taking into account the unique combination of characteristics for each patient.

The performance of the clinical-radiomics nomogram was evaluated using various statistical measures. Calibration curves were constructed to assess the agreement between the predicted probabilities from the nomogram and the actual probabilities observed in the data, aiming for a close match to the 45° line indicating perfect calibration. The nomogram's discriminative ability was evaluated using AUC, with values closer to 1.0 denoting superior discriminatory ability. Additionally, decision curve analysis was performed to evaluate the clinical usefulness of the nomogram by assessing the net benefits at different threshold probabilities, helping to visualize the potential benefit of using the nomogram for decision-making across various risk thresholds.

### Statistical analysis

The statistical analysis was conducted utilizing the Deepwise DxAI platform (version 1.0.3, http://dxonline.deepwise.com). Descriptive statistics, including mean, variance, frequency, and percentage, were employed for comprehensive data characterization. Prior to hypothesis testing, an assessment of normality was performed on numerical variables. Subsequently, normally distributed variables were tested using independent sample t-tests, while non-normally distributed variables were tested using Wilcoxon tests. Using unordered categorical variables, the chi-square test was applied. Significance levels were determined using a two-tailed t-test, with a predetermined threshold of *P* < 0.05, denoting statistical significance.

### Ethical approval and consent

Ethical approval to access the patients data was granted by The Second Affiliated Hospital of Harbin Medical University (No. HSA2014-075).

## Results

### Clinicoradiological characteristics

Table 1 presents a comprehensive comparison of clinical and radiological features between the UC and CD groups. Gender distribution demonstrated no significant difference (*P* = 0.584), with comparable percentages of females and males in both groups. The assessment of histologic fibrosis revealed that the majority of patients in both groups exhibited none to mild fibrosis, and there was no significant disparity in fibrosis severity between the UC and CD groups (*P* > 0.05). Perienteric edema or inflammation, engorged vasa recta, and lymphadenopathy showed no significant differences between the UC and CD groups (all *P* > 0.05). Lesion location analysis indicated no significant variation between the groups (*P* = 0.101), with the colon and cecum being the most common locations in both. Additionally, there were no significant differences in age and thickness of the intestinal wall (all *P* > 0.05). However, a significant difference was observed in AP-CT value between the UC and CD groups (*P* < 0.05).

**Table 1:** The characteristics of patients with inflammatory bowel disease.

Features	UC (n = 105)		CD (n = 113)		*P* value
	n	%	n	%	
Gender					0.584
Female	50	47.62%	58	51.33%	
Male	55	52.38%	55	48.67%	
Histologic fibrosis					0.880
None–mild	64	60.95%	70	61.95%	
Moderate–severe	41	39.05%	43	38.05%	
Perienteric edema or inflammation	63	60.00%	65	57.52%	0.710
Engorged vasa recta	46	43.81%	53	46.90%	0.647
Lymphadenopathy	58	55.24%	64	56.64%	0.835
Lesion location					0.101
Terminal ileum	28	26.67%	21	18.58%	
Cecum	29	27.65%	31	27.43%	
Colon	37	35.24%	36	31.86%	
Rectum	11	10.48%	25	22.12%	
Age (years)	46.55 ± 13.28		47.05 ± 16.33		0.864
Thickness of intestinal wall (mm)	9.55 ± 2.71		11.00 ± 3.28		0.382
AP-CT value (Hu)	53.94 ± 17.58		65.57 ± 14.25		0.034*

*P < 0.05.

*CD* Crohn’s disease, *UC* ulcerative colitis, *AP-CT value* CT value of arterial phase-enhancement.

### Radiomics score building

Table 2 summarizes the performance of different models on the training set (n = 145) and test set (n = 73). The models include LDA, LR, RF, SGD, SVM, clinical model, and the nomogram. On the test set, the nomogram demonstrated the highest AUC of 0.865 (95% CI 0.738–0.992), indicating excellent discriminative ability. It also exhibited a high accuracy of 0.791, sensitivity of 0.852, and specificity of 0.913. The LR model achieved the second-highest AUC of 0.821 (95% CI 0.647–0.995) with a high accuracy of 0.656 and sensitivity of 0.812. The SVM model had the lowest AUC of 0.711 (95% CI 0.559–0.863) and moderate performance metrics. On the training set, the nomogram maintained a high AUC of 0.971 (95% CI 0.950–0.992) and achieved exceptional accuracy, sensitivity, and specificity. The RF and SVM models exhibited perfect AUCs of 1.000, indicating excellent performance. The other models, including LDA, LR, and SGD, demonstrated good discriminative ability with AUC values ranging from 0.922 to 0.988. Overall, The LR model demonstrated superior performance on the test set, while all models exhibited excellent performance on the training set.

**Table 2:** Performance of different models on the training set and test set.

Model	Test set (n = 145)				Training set (n = 73)			
	AUC (95% CI)	Accuracy	Sensitivity	Specificity	AUC (95% CI)	Accuracy	Sensitivity	Specificity
LDA	0.738 (0.589–0.907)	0.691	0.834	0.528	0.922 (0.909–0.935)	0.828	0.879	0.728
LR	0.821 (0.647–0.995)	0.656	0.812	0.517	0.975 (0.963–0.987)	0.972	0.962	0.958
RF	0.718 (0.535–0.901)	0.635	0.800	0.319	1.000 (0.998–1.000)	1.000	1.000	1.000
SGD	0.817 (0.646–0.987)	0.608	0.729	0.525	0.988 (0.977–0.999)	0.945	0.962	0.913
SVM	0.711 (0.559–0.863)	0.619	0.631	0.628	0.997 (0.992–1.000)	0.986	0.974	1.000
Clinical model	0.602 (0.447–0.757)	0.563	0.693	0.517	0.735 (0.716–0.754)	0.689	0.619	0.732
Nomogram	0.865 (0.738–0.992)	0.791	0.852	0.913	0.971 (0.950–0.992)	0.996	0.824	0.965

*LDA* linear discriminative analysis, *LR* logistic regression, *RF* random forest, *SGD* stochastic gradient descent, *SVM* support vector machine.

In the LR model, the top 10 features with the highest weights were carefully selected. A probability score, called a Rscore, is generated by calculating correlation coefficients between selected features and outcomes. It served as a representative measure for assessing the risk of fibrosis for IBD. The formula was as follows:

$$\begin{gathered} {\text{Radiomic score}} \hfill \\ \quad \quad = 0.36732{\text{*wavelet}} - {\text{HHL}}\_{\text{glszm}}\_{\text{GrayLevelVariance}} + 0.4281 \hfill \\ \quad \quad {\text{*wavelet}} - {\text{LLL}}\_{\text{glcm}}\_{\text{Imc}}2 + 0.2896 \hfill \\ \quad \quad {\text{*exponential}}\_{\text{glrlm}}\_{\text{ShortRunEmphasis}} + 0.1778 \hfill \\ \quad \quad {\text{*logarithm}}\_{\text{gldm}}\_{\text{LargeDependenceLowGrayLevelEmphasis}} + 0.3584 \hfill \\ \quad \quad {\text{*squareroot}}\_{\text{glszm}}\_{\text{LargeAreaEmphasis}} - 0.2987{\text{*wavelet}} \hfill \\ \quad \quad - {\text{HHH}}\_{\text{firstorder}}\_{\text{Median }} - 0.3012 \hfill \\ \quad \quad {\text{*original}}\_{\text{shape}}\_{\text{Maximum}}2{\text{DDiameterSlice}} - 0.2883 \hfill \\ \quad \quad {\text{*gradient}}\_{\text{firstorder}}\_{\text{Minimum}} - 0.3841{\text{*original}}\_{\text{shape}}\_{\text{Sphericity}} \hfill \\ \quad \quad - 0.3356{\text{*gradient}}\_{\text{glrlm}}\_{\text{RunLengthNonUniformity}} \hfill \\ \end{gathered}$$

### Clinical-radiomics nomogram building and validation

**Figure 2:**
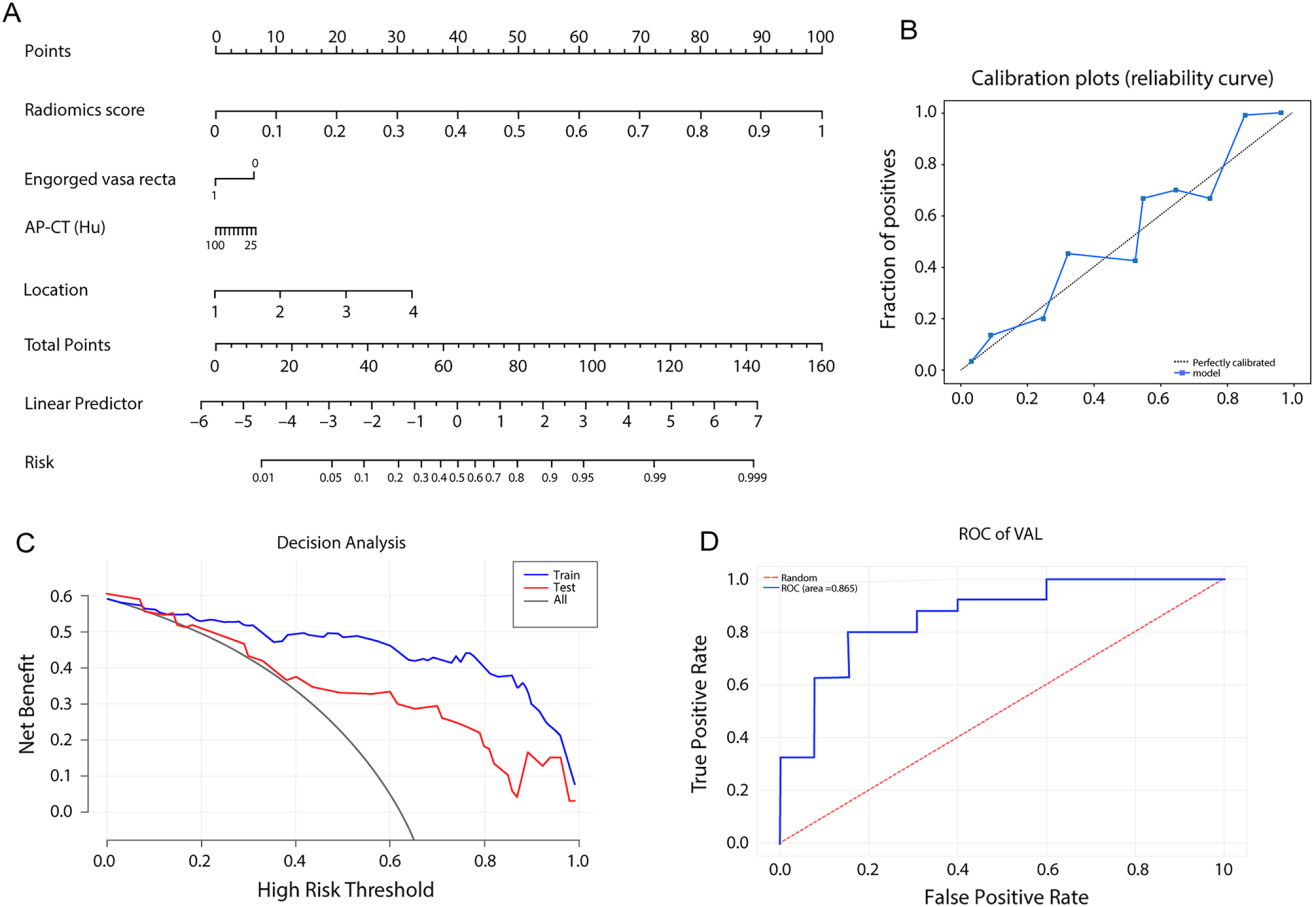
The construction of a personalized comprehensive nomogram and the assessment of its performance in predicting the risk of fibrosis in inflammatory bowel disease. (**A**) Nomogram; (**B**) calibration curve; (**C**) decision curve; (**D**) ROC curve on the test set. ROC, receiver operating characteristic.

**Figure 3:**
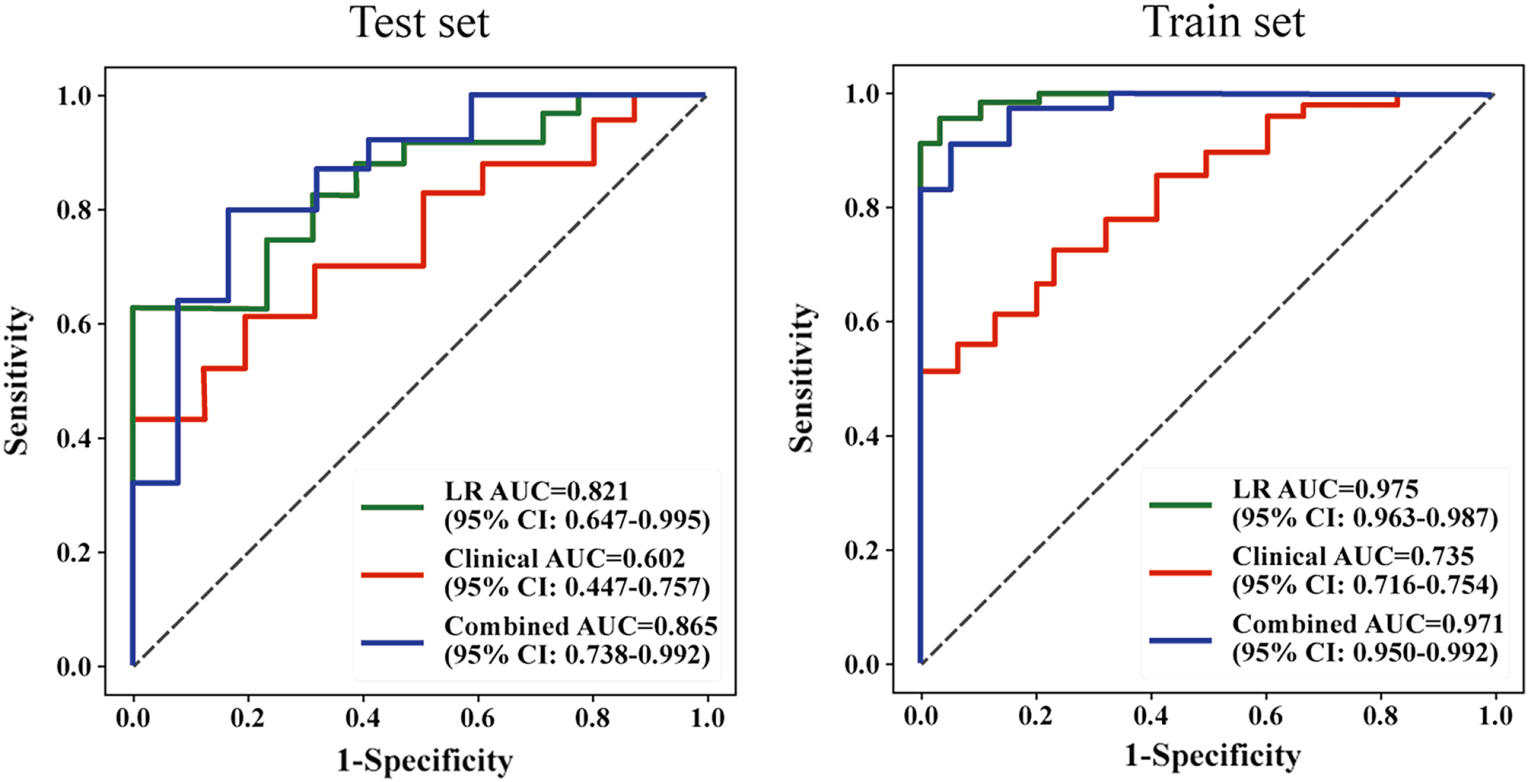
ROC curves illustrate the performance of the LR model, clinical model, and clinical-radiomics model on the training set and the validation set.

In the construction of the nomogram by combining the radiomics features and clinical factors, the radiomics score, engorged vasa recta, AP-CT value, and lesion location were integrated as predictive factors (Fig. 2A). Each factor was assigned a specific point value based on its relative contribution to the overall risk assessment. By summing the points associated with each factor, a total point score was calculated, which was then translated into a predicted probability of the fibrosis of IBD using a calibration curve (Fig. 2B). The model performance on the training set and test set was shown in Fig. 2C by a decision curve. In the validation sets, the AUC of the ROC curve, a widely used metric for evaluating diagnostic accuracy, was determined to be 0.865 (Fig. 2D). This indicates a favorable discriminative ability of the nomogram in distinguishing the fibrosis of IBD. We also compared the performance of different models in both the training set and the test set (Fig. 3). In the test set, the LR model exhibited an AUC of 0.821, indicating good predictive performance. On the other hand, the clinical model achieved an AUC of 0.602, suggesting lower discriminatory ability. Notably, the nomogram demonstrated the highest AUC of 0.865, indicating superior predictive accuracy compared to both LR and the clinical model. In the train set, the LR model achieved a high AUC of 0.975, surpassing the clinical model with an AUC of 0.735. Similarly, the nomogram showcased excellent performance with an AUC of 0.971, further highlighting its predictive superiority over the other models.

## Discussion

Accurate measurement of fibrosis in IBD is essential for the effective management and prognosis of patients. However, conventional approaches suffer from limitations, particularly subjective interpretation by radiologists, leading to variability and potential diagnostic errors. Therefore, there is a pressing need to improve fibrosis assessment in IBD. To address these limitations, this study focuses on leveraging the emerging field of radiomics, which combines advanced imaging techniques and computational algorithms to extract quantitative features from medical images. By utilizing radiomics, the study aims to develop a novel radiomics nomogram that integrates MSCT images and clinical factors. This nomogram holds great promise in providing more objective and accurate fibrosis assessment in IBD patients. The ultimate goal is to enhance clinical decision-making and improve patient care by overcoming the subjectivity and variability associated with conventional approaches.

The development of fibrosis in IBD involves a complex interplay of various factors, including chronic inflammation, extracellular matrix remodeling, and profibrotic signaling pathways^30,31^. Persistent inflammation triggers the activation of fibroblasts, which then produce excessive collagen and other extracellular matrix components, leading to the formation of fibrotic tissue^32^. MSCT imaging provides high-resolution images and allows for multi-planar reconstruction, enabling the visualization of morphological features, bowel wall thickness, and lesion distribution. MSCT can also evaluate vascular supply and vasodilation of the intestines, which are important factors in the development of fibrosis. In MSCT examinations, findings such as increased bowel wall thickness, luminal stenosis, and mural stratification are indicative of fibrosis^33,34^. Moreover, the presence of engorged vasa recta and pericentric fat stranding may suggest the severity of fibrosis^35^. MSCT not only aids in the detection and localization of fibrotic lesions but also assists in assessing the extent and complications associated with fibrosis, such as strictures and fistulas^36^. The use of MSCT in fibrosis evaluation in IBD offers several advantages, including its non-invasive nature, widespread availability, and ability to provide detailed anatomical information^12^. In this study, a nomogram was developed to predict fibrosis in IBD by integrating radiomics score, engorged vasa recta, AP-CT value, and lesion location. The nomogram demonstrated superior predictive accuracy with an AUC of 0.865 in the validation sets, outperforming logistic regression and clinical models. This result can primarily be attributed to the nomogram's integrated approach, which synergistically combines clinical data with radiomic features. This comprehensive framework harnesses the strengths of both data types, offering a more nuanced and holistic assessment than models relying on singular data sources. The integration of diverse data types potentially captures a broader spectrum of disease markers, resulting in improved predictive accuracy. These findings highlight the potential of the nomogram as a valuable tool for accurately stratifying fibrosis in IBD patients.

In contrast, the LR model, which achieved the second-highest AUC of 0.821 (95% CI 0.647–0.995), may have been limited by its focus on a singular type of data (only radiomic). This limitation could account for its slightly lower performance compared to the nomogram. The SVM model displayed the lowest AUC of 0.711 (95% CI 0.559–0.863). The underperformance of the SVM model might be attributed to its inherent characteristics, such as sensitivity to the scale and distribution of the data, which may not have been ideally suited for the heterogeneous nature of our dataset^37^. The SVM model's moderate performance emphasizes the necessity of choosing appropriate machine learning algorithms that align with the specific attributes of the data being analyzed. The similar results can also be found in previous studies^34^.

Additionally, the number of features selected in a model crucially impacts its accuracy, risk of overfitting or underfitting, computational cost, interpretability, and the potential inter-correlations among features, thereby influencing the overall effectiveness and efficiency of the model^38^. In our study, the meticulous selection of the top 10 features, characterized by their significantly higher weights, was informed by their proven relevance to IBD fibrosis. These features were not only chosen for their superior predictive power but also for their substantial contribution to the model's overall accuracy. This selection process reflects a comprehensive and intentional effort to identify the most informative and relevant radiomic characteristics. Consistent with existing literature, this approach employs image-based radiomics signatures to proficiently differentiate between disease and control groups, underscoring the method's effectiveness in disease characterization^39,40^.

Most interestingly, engorged vasa recta, AP-CT value, and lesion location are important factors in the assessment of fibrosis in IBD due to their significant contributions and clinical value. Engorged vasa recta reflect the degree of vascular supply and vasodilation in the intestines, which are closely associated with the development of fibrosis^41^. This feature provides valuable information about the extent and severity of fibrotic changes in the bowel wall. The AP-CT value, obtained through MSCT, is a quantitative measure that reflects the density of tissues. In the context of fibrosis, a higher AP-CT value indicates increased collagen deposition and fibrotic tissue, allowing for the identification and characterization of fibrotic lesions^42^. This parameter offers an objective and quantitative assessment of fibrosis severity, aiding in treatment planning and monitoring disease progression. Lesion location is another crucial factor in evaluating fibrosis in IBD. The specific site and distribution of fibrotic lesions provide insights into the spatial pattern and involvement of different segments of the gastrointestinal tract. This information helps in determining the extent and complications associated with fibrosis, such as strictures and fistulas^43^. Incorporating lesion location into the nomogram enables a more comprehensive assessment of fibrosis and assists in personalized treatment decisions.

Despite its significant findings, this study has a few limitations. Firstly, the sample size in the study was relatively small, which may limit the generalizability of the findings. A larger sample size would provide more robust results and enhance the reliability of the nomogram. Secondly, the study mainly focused on MSCT and clinical factors, potentially overlooking other relevant variables that could influence fibrosis in IBD. Including a broader range of factors, such as genetic markers or histopathological characteristics, would provide a more comprehensive understanding of fibrosis in this context. Thirdly, although the performance of the radiomics nomogram was promising, external validation in independent cohorts is necessary to confirm its accuracy and generalizability. Lastly, the study did not explore the impact of treatment interventions or longitudinal changes in fibrosis over time, which could provide valuable insights into disease progression and therapeutic response. Future research should address these limitations to further strengthen the clinical applicability and utility of the radiomics nomogram in stratifying fibrosis in IBD. In considering the implementation of the radiomics nomogram in clinical settings, the integration of this tool holds great promise for enhancing patient care, yet several potential barriers and considerations must be addressed to ensure its widespread adoption. Standardizing imaging protocols and ensuring data consistency are crucial for reliable results, while addressing data privacy and security concerns is essential to maintain patient confidentiality. Additionally, fostering interdisciplinary collaboration and ensuring the medical staff’s proficiency with the tool are vital steps towards seamlessly incorporating the radiomics nomogram into routine clinical practice.

In conclusion, the radiomics nomogram based on MSCT and clinical factors shows promise in stratifying fibrosis in inflammatory bowel disease. It outperforms traditional clinical models and provides a personalized risk assessment. Further validation and addressing identified limitations are needed to enhance its applicability. Implementing this nomogram can improve patient care by enabling accurate fibrosis stratification and guiding tailored treatment strategies in IBD.

## Data Availability

The datasets used and/or analysed during the current study available from the corresponding author on reasonable request.
