# Author Correction: A radiomics nomogram based on MSCT and clinical factors can stratify fibrosis in inflammatory bowel disease

**DOI:** 10.1038/s41598-024-56450-2

**Published:** 2024-03-13

**Authors:** Xu Zeng, Huijie Jiang, Yanmei Dai, Jin Zhang, Sheng Zhao, Qiong Wu

**Affiliations:** Department of Radiology, The Second Affiliated Hospital of Harbin Medical University, No. 246 Xuefu Road, Nangang District, Harbin, 150081 Heilongjiang Province People’s Republic of China

Correction to: *Scientific Reports* 10.1038/s41598-023-51036-w, published online 12 January 2024

The original version of this Article contained an error in the Methods section, under the subheading ‘Image data’.

“For the enhanced scanning procedure, a SOMATOM definition flash CT machine was used.”

now reads:

“For the enhanced scanning procedure, 256-detector rows CT machine was used.”

Additionally, in the Reference standard for intestinal fibrosis section, under the subheading ‘Feature selection and radiomics score construction’.

“To ensure proper model evaluation, the dataset was divided into training (n = 279) and testing (n = 139) sets.”

now reads:

“To ensure proper model evaluation, the dataset was divided into training (n = 145) and testing (n = 73) sets.”

The original Article has been corrected.

